# Health Shocks and Household Education Burden—A Study From the Perspective of Relative Poverty Alleviation in China

**DOI:** 10.3389/fpubh.2022.877052

**Published:** 2022-04-27

**Authors:** Zhenyu Li, Xinghua Wang, Yuning Chu

**Affiliations:** ^1^School of Economics, Qingdao University, Qingdao, China; ^2^Normal College, Qingdao University, Qingdao, China; ^3^Department of Nutrition, The Affiliated Hospital of Qingdao University, Qingdao, China

**Keywords:** health shocks, household education burden, peer effects, relative poverty, alleviation

## Abstract

Health shocks and household education burden influence levels of expenditure on healthcare and education, which are two major non-discretionary expenditures for households. From the perspective of relative poverty alleviation in China and based on the peer effects theory, this study uses the dataset from the rural areas in CFPS database and employs the spatial Durbin model and spatial DID model to investigate—when a household suffers health shocks—the influence of such impact on the education burden of closely related households and to test the effect of single rescue policy in this circumstance. Further, this study employs a spatial mediating effect model to analyze the spatial transmission mechanism. The results indicate that when a household has health shocks, it can aggravate the education burden of closely related households through inter-household social networks. The findings substantiate that the targets of different rescue policies have cross effects and that single rescue policy does not have significant effect on the targets of other policies. To avoid the situation where rescue policies operate in silos and to reduce the internal coordination cost between different policies within a system, a coordinating mechanism should be established between different rescue policies to better alleviate relative poverty.

## Introduction

Poverty and anti-poverty are among the major challenges that are facing today's world. As of the end of 2020, China had achieved the goal of comprehensively eliminating absolute poverty. In the future, the focus of the battle against poverty will shift from “absolute poverty” to “relative poverty”^1^. Different from absolute poverty, which is measured by the monetary value of food consumption, relative poverty relates to “relative deprivation” and is reflected through inequity in income allocation and access to public services and through lower levels of education, healthcare, senior care, and social security (1, 2). In comparison with absolute poverty, relative poverty is ever-developing, multidimensional, structured, and related to special groups (3). Existing study has indicated that there are significant differences in the measures required to alleviate the two types of poverty. Relative poverty has broader implications and much larger coverage; identifying people living in relative poverty requires not only consideration of the income dimension, which is the focus of absolute poverty, but also consideration of the needs (or expenditure) dimension, which is unique to relative poverty (4). As a result, previous approaches to alleviating absolute poverty, which mainly used income level to assess poverty, did not necessarily have a one-to-one corresponding relationship with targets of relative poverty alleviation and may have seriously underestimated the extent of poverty (5) and undermined the sustainability of poverty alleviation policies (6). The impact of relative poverty on individual development tends to last for a long time (7). Poverty alleviation through the social security system plays a more important role than does development-oriented poverty alleviation (8). As such, the government should ensure that a needs-oriented social security system is in place, focusing more on expenditures on education, healthcare, and housing, which are of critical significance to people's rights to subsistence and development (9, 10), and developing equitable public policies that are favorable to people living in relative poverty (11).

Based on the above discussion, this study is of the view that relative poverty alleviation should focus more on the sustainability of providing support through public finance. To avoid the situation in which rescue policies operate in silos and are not connected with one another, there is a need to develop a mechanism that coordinates various rescue policies or different social security programmes so that a comprehensive synergistic effect can be achieved.

In developing a coordinating mechanism, first, the government should investigate and test whether the targets of different rescue policies influence one another, as well as the mechanism by which the influences occur; this will serve as a foundation for the various rescue policies to achieve a coordinated effect. Existing study does not provide a definite conclusion on this issue. Certain studies have examined changes in households' decisions on spending when they experience an external shock and investigated the effect of single rescue policy. Take studies of the impact of health shocks on households' spending decisions as an example. Most studies find that the increased healthcare expenditures as a result of household members experiencing health shocks inevitably affect various aspects of household life, including the time allocation between work and leisure (12), consumption-savings ratio (13), and preference for investment risks (14), forcing the household to adjust their behaviors in making economic-related decisions (15) and ultimately creating a significant crowding-out effect on other household expenditures, such as income, labor supply, and education (16). This effect is more evident in rural households and households with a medium-level income (17, 18). Further, when an individual household member suffers health shock, the shock will spread within the entire household (19) and indirectly affect the economic life of other household members, thereby increasing the probability that the entire household will fall into relative poverty (20). Studies have found that fiscal expenditures on rescue policies, especially increased fiscal expenditures on social programmes, such as subsidies for healthcare and education for low-income residents, are more effective in alleviating relative poverty in rural areas than are development-oriented fiscal expenditures (21); this is because the former has a more significant effect on raising the cost of labor supplied by the poverty-stricken population (22).

Existing study has mainly focused on changes in spending decisions within households that have experienced health shocks but fall short of examining the impact of spending decisions made by the household in question on other households via the social networks between households. As a result, in alleviating relative poverty in the future, there might exist a connection cost between individual rescue policies that each aim at a single target; in other words, because a rescue policy focuses on one public service area, the impact of the policy is limited. Further, there may be an interactive effect between different rescue policies, and the effect of multiple overlapping rescue policies is unknown; this may cause issues such as the inaccurate identification of people in relative poverty and the low sustainability of poverty alleviation policies.

In summary, this study proposes that it is necessary to reduce the scope within which the identification of people in poverty is conducted. By examining how household spending decisions affect one another in a social network as well as the mechanism by which the influences occur, we can reveal the patterns of behaviors of special groups and therefore overcome the challenge of identifying people in relative poverty. Furthermore, we should take into full consideration the complex consequences of a rescue policy and, by developing a coordinating mechanism between rescue policies, effectively alleviate relative poverty. In view of this, this study uses the dataset from the rural areas in the China Family Panel Studies (CFPS) database and two major non-discretionary household expenditures—healthcare and education—as examples to investigate the two major issues. First, this study employs the spatial Durbin model and spatial difference in differences (DID) model to investigate—when a household suffers health shocks—the influence of the event on the education burden of other households that are close to the household in question and to test the effect of an existing single rescue policy in this circumstance. Second, this study employs the spatial mediating effect model to analyze the transmission mechanism between the health shocks and household education burden.

This study carries the following contributions. In terms of the perspectives, this study takes the peer effects as theoretical basis, and explores the feasibility of establishing a coordinating mechanism. The research findings are helpful to improve the accuracy of identifying relatively poverty objects, eliminate the inequality and inequity of rescue policy objects in policy acquisition. With regard to the contents, the analysis of the impact of education and other household expenditure after household suffering from health shocks is no longer limited to a single household. It extends to the spatial dimension. The empirical tests in this study can help develop a coordination mechanism between healthcare rescue policies and education rescue policies so that the rescue policies operating in silos can be eliminated and the connection costs between different policies within a system can be reduced. In terms of the methods, the research models and analysis methods used in this study clearly cover three aspects of the research topic, namely, the correlation between target variables, the transmission mechanism and the effectiveness of current policies. It has laid a foundation for building a systematically integrated, effective, and multi-level social security system that will improve relative poverty alleviation.

## Research Design

### Theoretical Basis and Research Hypothesis

China is a typical relationship-based society where people frequently interact with each other. As a result, an individual's behaviors are subject to the influence of the behaviors of surrounding groups that have similar characteristics (23); in other words, peer effects significantly affect people's behaviors (24). Peer effects are considered externalities that spill over from peers' characteristics and behaviors (25). Research has verified that peer effects exist in many aspects, such as children's behavior, household investment and consumption decision-making and so on (26, 27). Neighbors and relatives may directly affect the skills, information, and social opportunities of other members of the society who are, to a certain extent, close to these households (28, 29). In this case, when a household suffers health shocks, the event may not only widen the gap in wellbeing between the household experiencing health shocks and those in the same social space not experiencing such shocks, but also increase the probability that similar groups in the same social space will fall into relative poverty, resulting in the spatial transmission effect of poverty caused by health shocks. Despite the growing volume of research on the impact of peer effects on the behaviors of individuals and households, the issue above has not been empirically tested in previous research.

**Figure 1:**
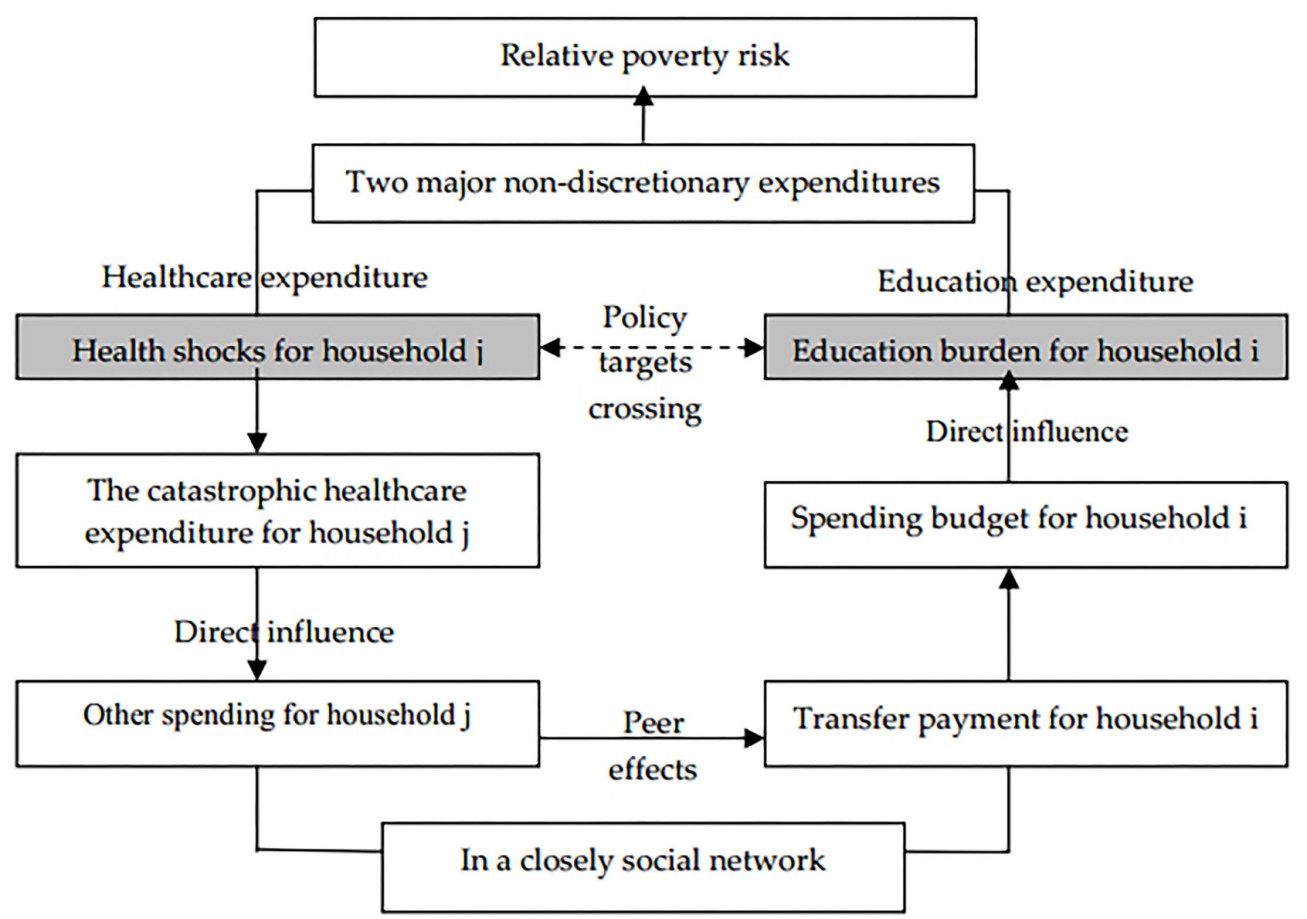
The logic of spatial transmission process between health shocks and household education burden.

The logic of this spatial transmission process is presented in Figure 1, as follows. A serious disease first changes the spending structure of the patient's household *j*, and the urgent health expenditures crowd out other household spending. When the total health expenditure exceeds the household *j*'s total wealth, the household *j* may ask for help from relatives, friends, and neighbors, such as household *i*. This amounts to redistributing the economic risk caused by the health shocks to other households in the social network and reducing the consumption budget constraints of the households offering help, thereby increasing their economic burden. If the social network of the patient's household lacks the ability to bear such economic burdens, the health shock is highly likely to increase the risk that other households in the network will fall into relative poverty, resulting in the spatial transmission of relative poverty via the social network. Here, we focus on the spatial relativity of relative poverty.

Based on the analyses above, this study puts forward the following three hypotheses:

H1: Health shock not only affects the consumption decision-making of household in question, but also affects the consumption decision-making of households with similar characteristics to a certain extent.

H2: The possible transmission mechanism of this impact depends on the social network, which is completed in the form of transfer payments between closely related households.

H3: The effect of single rescue policies on the targets of other rescue policies is not clear.

### Methods and Variable Selection

To test the above hypotheses, this study utilizes the spatial Durbin model, and constructs a spatial DID model and spatial mediating effect model, respectively, to empirically analyse—when a household suffers health shocks—the influence of such impacts on the education burden of closely related households and the effect of single rescue policy in this circumstance. It also analyses the influencing mechanism between the health shocks and household education burden that form through transfer payments between households.

The advantages of selecting the above models are as follows. First, the spatial econometric model has been widely adopted in analyzing social interactions between individual economic entities (30). Together, the spatial lag model, spatial error model, and spatial Durbin model constitute a spatial econometric model system. The spatial Durbin model not only can represent the impact of the core explanatory variables of the closely related households on the explained variables of the household in question, but also quantify the effects of other explanatory variables. Considering the distribution characteristics of the explained variables, this study adopts the spatial Tobit Durbin model to describe the impact on the household education burden after a closely related household suffers health shocks. Second, while the analysis of the effect of the integrated urban and rural medical insurance (IURMI) system performed using the spatial DID model can represent changes in household education burden in regions that first adopt the policy, it further reflects peer effects in household education burden in these regions. In addition, compared with the regular DID model, the spatial DID model can produce more consistent estimates; the greater is the spatial autocorrelation coefficients of the dependent variables, the more valid is the policy effect estimated by the spatial DID model (31). Third, with regard to the path through which the health shocks influence the household education burden of closely related household, based on the spatial Durbin model, the study draws from the mediating effect model of Wen et al. (32) to construct a spatial mediating effect model.

In summary, in the first part of the empirical analysis, the spatial Durbin model (model 1) and the spatial DID model (model 2) are constructed, as follows:


$$\begin{array}{l} EDU_{i}=\rho wEDU_{j}+\beta_{1}CHE_{i}+\delta_{1}wCHE_{j}+\beta X\\ \;+\delta wX+\mu \;(\text{model}\;\text{1})\\ EDU_{i}=\rho wEDU_{j}+\gamma_{1}T^{*}Treated_{i}+\gamma_{2}wT^{*}Treated_{j}+\beta_{1}CHE_{i}\\ \;+\delta_{1}wCHE_{j}+\beta X+\delta wX+\mu \;(\text{model}\;2) \end{array}$$

In model (1), the explained variable *EDU*_*i*_ denotes the education burden of household *i*, which is measured by the household's total education expenditure as a proportion of its total disposable income within the year, and its value is between 0 and 1. The core explanatory variable *CHE*_*j*_ is the catastrophic healthcare expenditure of closely related household *j*. The definition of catastrophic healthcare expenditure is based on the criteria of the World Health Organization (WHO); if, during the past 12 months, the ratio between the household's healthcare expenditure and the household's total expenditure, minus expenditures on food, is higher than 40%, then the household is deemed to have incurred a catastrophic healthcare expenditure and suffered health shocks, and the variable is assigned a value of 1; otherwise, it is assigned a value of 0. *w* denotes the spatial geographic weight matrix factor, namely, the distance between households in the sample. Rural villages in China are usually distributed based on family lines; households in the same village tend to belong to the same family tree and residents have the same family name. The shorter is the distance between households, the closer is the blood-based relations between the households, and the greater is the possibility that households will provide economic support to each other. Considering the statements above, this study uses “whether the households belong to the same village” to construct the spatial weight matrix. ρ denotes the spatial correlation between household education burden; when ρ < 0, it is deemed there is a spatial substitute effect between the education burdens of closely related households; when ρ > 0, there is a spatial spillover effect between the education burdens of closely related households; and when ρ = 0, there is no spatial effect between the education burdens of closely related households. Control variable *X* represents the household income level, preferred object for borrowing, whether the household receives government subsidies, the degree of the household's participation in social medical insurance, and whether the household has a mortgage. μ denotes the error.

To further investigate the effect of an existing single rescue policy interacting with the goals of other policies, model (2) introduces a spatial DID model to test the impact of two social security policies—the IURMI policy—on household education burden. The main content of the IURMI policy is the integration of the New Rural Cooperative Medical Scheme with the Urban Resident Basic Medical Insurance Scheme; the goal of this policy is to ensure that rural residents in regions where the policy is implemented have access to basic medical insurance that is comparable to that provided to urban residents, thereby increasing the medical insurance level of rural residents. In practice, as the time when the IURMI policy was implemented differs among China's provinces, in referencing the approach described by Ma et al. (33), this study selects Chongqing (began implementation in 2008), Tianjin (began implementation in 2010), and Guangdong (began implementation in 2012), which were the first jurisdictions to implement the policy, as the treatment group; the other provinces (gradually began implementation after 2016) constitute the control group. Considering the lag of policy effect, the CFPS datasets for 2014 and 2018 are used as the pre-test and post-test data, respectively, to analyze the policy effect. Building on this, this study further incorporates into the model whether a household receives a general fiscal subsidy from the government, so as to examine the combined effect of the IURMI policy and general fiscal government subsidies. The general fiscal subsidy refers to the government's transfer payments made to low-income groups and people experiencing difficulties in everyday living to ensure that these groups can afford normal subsistence expenses. In model (2), *Treated* denotes whether a household in a province has implemented the IURMI policy. *T* denotes the point in time at which the IURMI policy began to demonstrate effects. The policy effects are measured based on whether γ_1_, the coefficient of T^*^Treated_*i*_, is significant. If γ_1_ is negative and significant, the IURMI system or general fiscal subsidies are effective in reducing household education burden; if γ_2_ is negative and significant, the policies' influences on household education burden have peer effects. The definitions of the other variables in model (2) are the same as those in model (1).

In the second part of the empirical analysis, the following spatial mediating effect model is constructed to test the spatial transmission mechanism. At stage 1, we perform a regression analysis for the catastrophic healthcare expenditure of a closely related household *CHE*_*j*_ and the education burden *EDU*_*i*_ of household *i* using the spatial Durbin model. At stage 2, we perform a regression analysis of the catastrophic healthcare expenditure of a closely related household *CHE*_*j*_ and the net transfer payment *M*_*i*_ by the household *i* using the spatial Durbin model. At stage 3, we perform a regression analysis of the catastrophic healthcare expenditure of a closely related household *CHE*_*j*_, the net transfer payment *M*_*i*_ by the household *i*, and the education burden *EDU*_*i*_ of household *i* using the spatial Durbin model.


$$\begin{array}{l} EDU_{i}=\rho wEDU_{j}+\beta_{1}CHE_{i}+\delta_{1}wCHE_{j}+\beta X\\ \;+\delta wX+\mu \text{ (Stage 1)}\\ M_{i}=\rho wM_{j}+\beta_{1}CHE_{i}+\delta_{1}wCHE_{j}+\beta X\\ \;+\delta wX+\mu \text{ (Stage 2)}\\ {\text{EDU}}_{\text{i}}=\rho {\text{wEDU}}_{\text{j}}+\beta_{1}{\text{M}}_{\text{i}}+\delta_{1}{\text{wM}}_{\text{j}}+\beta_{2}CHE_{i}+\delta_{2}wCHE_{j}\\ \;+\beta \text{X}+\delta \text{wX}+\mu \text{ (Stage 3)} \end{array}$$

In the model, the explained variable *EDU* is the household education burden. The core explanatory variable *CHE* is the household's catastrophic healthcare expenditure. *M* is a mediating variable that is measured by whether the household has a net transfer payment over the past 12 months, namely, the difference between the total transfer payment the household made to other households at no cost and the total transfer payment the household received from other households at no cost. A positive value for *M* indicates a net transfer payment, and the variable is assigned a value of 1; otherwise, a value of 0 is assigned to the variable. During the three stages of the regression analysis, if the regression coefficients of the core explanatory variable at stage 1 and stage 2 and the mediating variable at stage 3 are all significant, then there is a mediating effect. ρ denotes the spatial correlation of the household education burden and the mediating variable; ρ < 0 indicates that there is a spatial substitute effect; ρ > 0 denotes that there is a spatial spillover effect, and ρ = 0 denotes that there is no spatial effect. The definitions of other variables are the same as those in model (1).

### Dataset

The CFPS datasets for 2014 and 2018 collected by the Institute of Social Science Survey of Peking University are used for this study for two main reasons. First, the datasets contain abundant information on households' expenditures on healthcare and education, transfer payments, and public service utilization. Further, the full implementation of the IURMI programme across China began in 2016; however, Chongqing, Tianjin, and Guangdong implemented the policy prior to that date. Therefore, the datasets for 2014 and 2018 provide appropriate information to analyze the influences of health shocks and the education burden and to analyze the spatial transmission mechanism of the influences, against the backdrop of the IURMI policy. Second, the data selected reflect three different micro levels (i.e., individual, household, and community) and provide a vivid picture of the changes in China's education, healthcare, and social welfare.

As the 2018 dataset does not contain a village code, in constructing the weight matrix, villages are matched with their codes in 2014. Further, records with missing information or inappropriate records within raw data were eliminated; therefore, a sample of 1,665 valid records was obtained.

## Results

### Descriptive Statistics

Table 1 presents the descriptive statistics of the 2018 dataset. Households' average level of education burden is 21.93%. As a non-discretionary expenditure, education spending accounts for a relatively high proportion of households' daily consumption, indicating that using household education burden to examine the spatial influence of households' spending decisions is reasonable. The probability of occurrence of catastrophic healthcare expenditure is 7.63%. 33.44% of households receive a net transfer payment. The average household income is 19,632.17 yuan (about 3,100 US dollars), and 57.29% of the households in the sample receive general fiscal subsidies. The high proportion of households receiving general fiscal subsidies indicates that when both the household education burden and catastrophic healthcare expenditure are considered together, the overall risk of relative poverty increases. Among the households in the sample, 82.52% participate in social medical insurance. Further, the standard deviation of each indicator is relatively large, an indication that currently, there are significant differences in the living conditions among rural households in China.

**Table 1:** Descriptive statistics.

**Variable**	**Mean**	**Standard deviation**	**Min**	**Max**
Household education burden	0.2193	0.2141	0	1
Catastrophic healthcare expenditure (occurrence is 1, non-occurrence is 0)	0.0763	0.2657	0	1
Net transfer payment (occurrence is 1, non-occurrence is 0)	0.3344	0.4720	0	1
Household income level (take logarithm)	10.3460	1.2967	2.5649	12.8212
Preferred object for borrowing (from 0 to 6, the objects are from banks and financial institutions to parents, relatives and friends. The larger the value, the closer the economic relationship is)	3.1979	2.0809	0	6
Household receipt of a fiscal subsidy (occurrence is 1, non-occurrence is 0)	0.5729	0.4949	0	1
Degree of participation in social medical insurance (expressed by the ratio of household insured persons to household size)	0.8252	0.3799	0	1
Mortgage (occurrence is 1, non-occurrence is 0)	0.1180	0.3228	0	1

### Impacts of Health Shocks on Household Education Burden

In order to verify the rationality of spatial Durbin model selected in this study, we first conducted Moran's I test and LM test. Moran's I test is widely used to verify the spatial correlation of variables and LM test provides suggestions for spatial model selections (34). Moran's I test results of household education burden and net transfer payment are −0.3215 (significant at 1% level) and −0.2852 (significant at 1% level), respectively. The result shows that two variables have spatial correlation. In Table 2, based on the LM test, the null hypothesis that there is neither spatial nor error dependence is rejected at the 1% level under all three models. In this case, the spatial Durbin model is superior to other spatial models (35). Next, we conducted Wald test. The results show that the Wald tests all reject the null hypothesis. It is reasonable to use the spatial Durbin model in terms of data.

**Table 2:** Regression results.

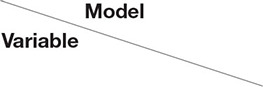	**Spatial Dobbin model**	**Spatial DID model**	**Spatial DID model**
	**(model 1)**	**(model 2)**	**(model 2)**
w* catastrophic healthcare expenditure	0.0017* (0.0010)	0.0031** (0.0012)	0.0038*** (0.0014)
w* household income level	0.0008*** (0.0002)	0.0013*** (0.0001)	0.0016*** (0.0001)
w* preferred object for borrowing	0.0003* (0.0002)	0.0002* (0.0001)	0.0003* (0.0001)
w* household receipt of a fiscal subsidy	−0.0001 (0.0007)	−0.0001 (0.0001)	
w* degree of participation in social medical insurance	−0.0007 (0.0008)		
w* mortgage	^−^0.0010 (0.0011)	0.0002 (0.0011)	0.00007 (0.0012)
T* Treated		^−^0.0012 (0.0024)	0.0008 (0.0008)
w* T		^−^0.0015** (0.0006)	^−^0.0015** (0.0007)
w* Treated		^−^0.0007 (0.0016)	^−^0.0036* (0.0019)
w* T* Treated		^−^0.0010 (0.0026)	0.0007 (0.0007)
ρ	^−^0.0014* (0.0007)	^−^0.0034*** (0.0007)	^−^0.0038*** (0.0008)
LM test	[0.0000]	[0.0000]	[0.0000]
Wald test	[0.0000]	[0.0000]	[0.0000]
Adjusted *R^2^*	0.5412	0.5304	0.5296

*(1) The brackets are heteroscedasticity robust standard errors*.

(2) ^*^, ^**^, and ^***^ are statistically significant at the significance levels of 10, 5, and 1%, respectively.

*(3) The result of LM test and Wald test are marked with p-value*.

**Figure 2:**
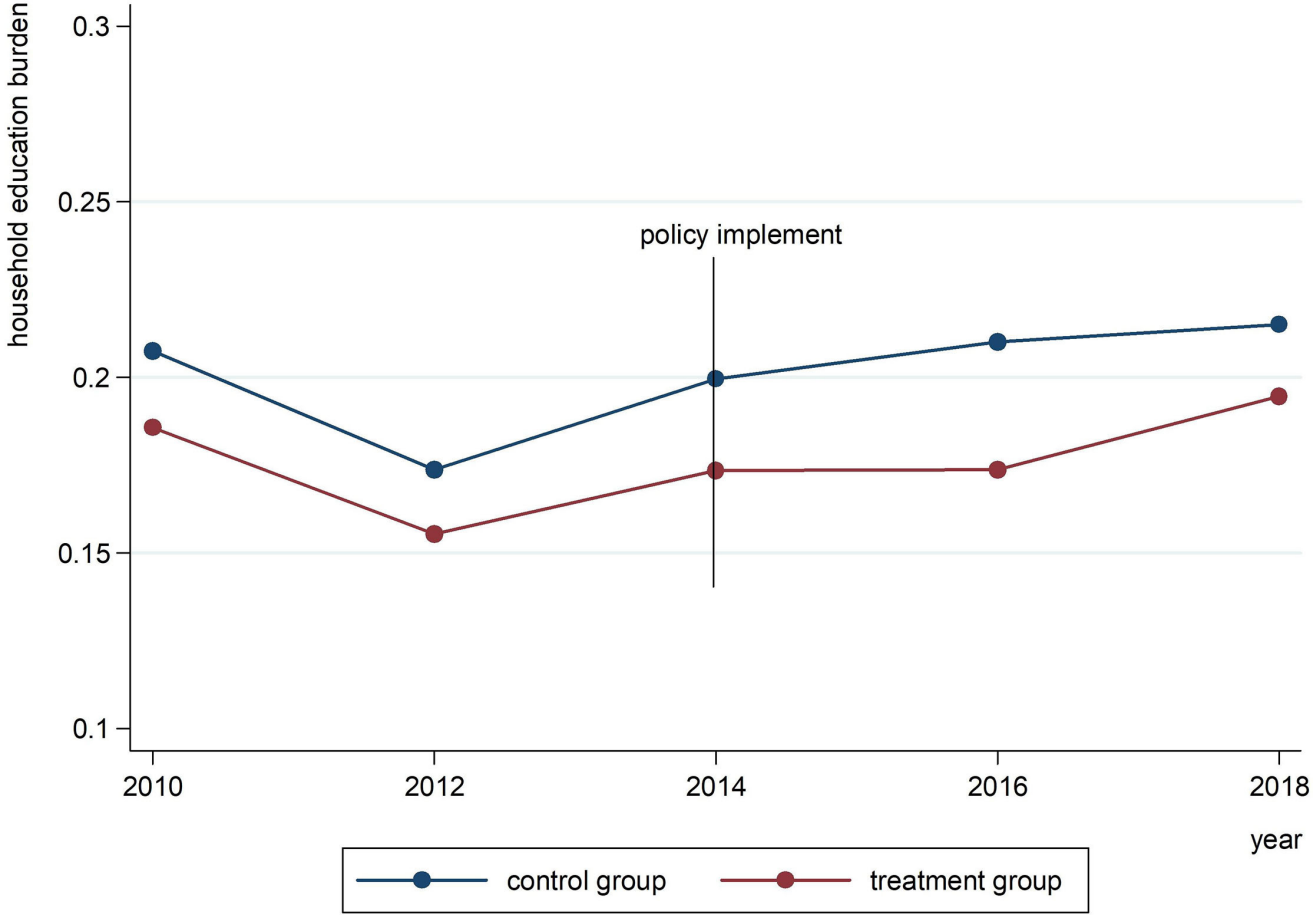
The parallel trend test.

In addition, we used the CFPS datasets over the years to carry out the parallel trend test. The dots in Figure 2 below represent the average level of household education burden in each year. Before the implementation of the IURMI policy, the trend of the treatment group is parallel to that of the control group between 2010 and 2014, and the average household education burden of the control group is higher than that of the treatment group, which may be related to the high level of economic development of Tianjin, Chongqing and Guangdong.

In Table 2, column 2 presents the results for the spatial Durbin model; column 3 presents the result for the spatial DID model in which adoption of the IURMI policy is the explanatory variable, and column 4 presents the result for the spatial DID model in which the interaction between two variables (i.e., adoption of the IURMI policy and the receipt of a fiscal subsidy) is the explanatory variable. The regression coefficient of w^*^catastrophic healthcare expenditure, which warrants special attention in all three models, is positive and significant at the 1, 5, and 10% levels; this finding indicates that when a household suffers health shocks, the catastrophic healthcare expenditure incurred by this household will significantly increase the education burden of closely related households, suggesting that currently, the two non-discretionary household expenditures—healthcare and education—influence each other. Further analysis indicates that neither variables w^*^household receipt of a fiscal subsidy and w^*^degree of participation in social medical insurance in model 1 nor the coefficients of T^*^Treated and w^*^T^*^Treated in model 2 and 3 are significant at the 10% level. This finding suggests that both the IURMI policy and the government's general fiscal subsidy do not significantly reduce household education burden. The above results indicate that the targets of different social security policies may have interactive influences on each other through specific spatial transmission mechanisms. Although single rescue policy (e.g., the medical insurance policy) may be effective with a single policy goal (e.g., a household's catastrophic healthcare expenditure), after considering the social network relations between households and the interactive influences between different policy targets, the original rescue policy does not have a spillover effect on other policy goals (e.g., household education burden). This conclusion further validates the importance of establishing the mechanism proposed by this study to coordinate different rescue policies.

The other variables have the same signs in the regression results under the three models; although certain variables are not significant, such results are consistent with the expected results. The coefficient of w^*^household income level is significant at the 1% level. This finding implies that the income level of closely related households is positively correlated with the household education burden and that the households are likely to bear the risk together; if the social network of the household has a fragile economic capacity, the health shocks will increase the risk that households in the social network as a whole may fall into relative poverty. w^*^preferred object for borrowing is significant at the 10% level, indicating the assumption that rural households decrease risk by borrowing from households in the same village is to a certain extent reasonable; the higher the tendency that the household borrows from parents, relatives, and good friends, the more likely it is that the household education burden will be aggravated. ρ is negative and significant, indicating that there is a substitution effect between household education burden and that close households in the same village may help each other with paying for education.

### Impact Mechanism Between Health Shocks and Household Education Burden

The above conclusions confirm that there are interactive spatial influences between health shocks and household education burden, but do not clarify the influencing mechanism. Table 3 employs the spatial mediating effect to analyze the mechanism at three stages.

**Table 3:** Regression results.

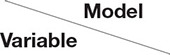	**Stage 1**	**Stage 2**	**Stage 3**
w^*^catastrophic healthcare expenditure	0.0017* (0.0010)	0.0006* (0.0003)	0.0036* (0.0019)
net transfer payment			0.0027* (0.0017)
w*household income level	0.0008*** (0.0002)	0.0004*** (0.0000)	0.0012*** (0.0004)
w*preferred object for borrowing	0.0003* (0.0002)	0.0001*** (0.0000)	0.0006*** (0.0003)
w* household receipt of a fiscal subsidy	^−^0.0001 (0.0007)	^−^0.0005** (0.0002)	0.0006 (0.0015)
w^*^degree of participation in social medical insurance	−0.0007 (0.0008)	^−^0.0011*** (0.0003)	^−^0.0014 (0.0016)
w* mortgage	^−^0.0010 (0.0011)	^−^0.0004 (0.0003)	^−^0.0020 (0.0021)
ρ	^−^0.0014* (0.0007)	^−^0.0005** (0.0002)	^−^0.0028* (0.0015)
LM Test	[0.0000]	[0.0000]	[0.0000]
Wald test	[0.0000]	[0.0000]	[0.0000]
Adjusted *R^2^*	0.5412	0.4981	0.5473

*(1) The brackets are heteroscedasticity robust standard errors*.

(2) ^*^, ^**^, and ^***^ are statistically significant at the significance levels of 10, 5, and 1%, respectively.

*(3) The result of LM test and Wald test are marked with p-value*.

For all three stages, the LM test and Wald test result rejects the null hypothesis at the 1% level, indicating that the spatial Durbin model is reasonable to use. The estimate of the impact of catastrophic healthcare expenditure on household education burden derived under the spatial Durbin model at stage 1 of the analysis is exactly the same as the result derived from model 1 in Table 2. At stage 2, the results of the spatial Durbin model that analyses catastrophic healthcare expenditures and households' net transfer payment indicate that w^*^catastrophic healthcare expenditure is positive and significant at the 10% level, indicating that if a household incurs a catastrophic healthcare expenditure, the probability that the household of closely related households will occur a net transfer payment will increase; that is, closely related households in the same village may borrow from each other. At stage 3, the results of the spatial Durbin model that analyses households' catastrophic healthcare expenditures, net transfer payments, and education burden indicate that w^*^catastrophic healthcare expenditure is still positive and significant at the 10% level, once again substantiating that the targets of the two rescue policies investigated by the study—healthcare and education—may affect each other. Further, net transfer payment is also positive and significant at the 10% level. The results of the three-stage regression analysis using the comprehensive mediating effect model confirm that when a household suffers health shocks and incurs a catastrophic healthcare expenditure, the economic impacts may increase the probability that the closely related households in the same village will occur a net transfer payment, and further change the consumption budget constraints and increase the education burden of closely related households. This transmission of economic impacts occurs via the inter-household social network when households provide economic support to each other.

The regression results for other variables in Table 3 essentially have the same signs as those in Table 2. ρ is negative at all three stages of the regression analysis, indicating that both household education burden and transfer payments have a spatial substitute effect. This finding indirectly substantiates that transfer payments are a mediating path through which households help each other pay for healthcare.

## Conclusions and Implications

### Conclusions

This study uses a dataset from rural areas in the CFPS database and two major non-discretionary household expenditures (i.e., healthcare and education) as examples to investigate the two major issues from the perspective of relative poverty alleviation. First, this study employs a spatial Durbin model and spatial DID model to investigate, when a household suffers health shocks, the influence of the impacts on the education burden of closely related households and to test the effect of an existing single rescue policy in this circumstance. Second, this study employs a spatial mediating effect model to analyze the transmission mechanism for the health shocks and household education burden. The results from this study indicate that in rural China, when a household suffers health shocks, the economic impacts may increase the probability that the closely related households in the same village will occur a net transfer payment and further change the consumption budget constraints and increase the education burden of closely related households via the inter-household social network when households provide economic support to each other. This conclusion indicates that the targets of the two rescue policies—healthcare and education—may influence each other. The results from this study further confirm that single rescue policy does not have a spillover effect on other targets and offer evidence that supports establishing a mechanism to coordinate different rescue policies.

### Implications and Policy-Making

In the future, to develop a systematically integrated, efficient, and multilevel social security system and achieve relative poverty alleviation, system designs should focus more on three aspects of governance coordination.

The first aspect is coordination between households with similar characteristics. The development of rescue policies should not be limited only to households that directly experience health shocks. The research conclusions of this study indicate that due to the existence of inter-household social networks, rescue policies should also consider households that have similar characteristics to those of the target households. A record system can be developed for similar households or communities to incorporate groups with similar characteristics into the scope of risk management, so as to overcome the challenges in identifying people in relative poverty and reduce the likelihood of region-wide relative poverty.

The second aspect is coordination between policy targets. The research conclusions of this study also indicate that the development of rescue policies should take into consideration the interactive influences between policy targets. For example, the targets of the education poverty alleviation policy should not only pertain to the population who are in educational poverty but should also incorporate the population in spending-based poverty. This approach will help to ensure that all policy goals are addressed in policies and to improve the effect of single rescue policies and the effect of relative poverty alleviation.

The third aspect is coordination between short-term and long-term rescue policies. When a household suffers from temporary external shocks, although single rescue policy may be effective in achieving a single policy goal of social security, after considering the inter-household social network and the interactive influence between policy goals, original rescue policies no longer generate significant effects regarding achieving other policy goals. Therefore, there is a need to establish a long-term sustainable rescue policy to eliminate other complications resulting from the interactions of policy goals.

## Data Availability Statement

Publicly available datasets were analyzed in this study. This data can be found here: [http://www.isss.pku.edu.cn/cfps/sjzx/gksj/index.htm].

## Author Contributions

ZL: conceptualization, methodology, formal analysis, and writing-original draft preparation. XW: formal analysis, writing-review, editing, and supervision. YC: resources, data curation, validation, and supervision. All authors have read and agreed to the published version of the manuscript.

## Funding

This study was funded by the National Social Science Fund of China (Nos.CFA200250).

## Conflict of Interest

The authors declare that the research was conducted in the absence of any commercial or financial relationships that could be construed as a potential conflict of interest.

## Publisher's Note

All claims expressed in this article are solely those of the authors and do not necessarily represent those of their affiliated organizations, or those of the publisher, the editors and the reviewers. Any product that may be evaluated in this article, or claim that may be made by its manufacturer, is not guaranteed or endorsed by the publisher.

## Footnotes

^1^The Fourth Plenary Session of the 19th Central Committee of the Communist Party of China called for “winning the tough battle against poverty and establishing long-term mechanisms for eliminating relative poverty”.
